# Development of a prediction model for the depression level of the elderly in low-income households: using decision trees, logistic regression, neural networks, and random forest

**DOI:** 10.1038/s41598-023-38742-1

**Published:** 2023-07-16

**Authors:** Kyu-Min Kim, Jae-Hak Kim, Hyun-Sill Rhee, Bo-Young Youn

**Affiliations:** 1 Department of Health Policy and Management, Graduate School, Korea University, Seoul, Korea; 2 BK21FOUR R&E Center for Learning Health Systems, Korea University, Seoul, Korea; 3 Department of Fitness Promotion and Rehabilitation Exercise, National Rehabilitation Center, Seoul, Korea; 4 Department of Health Policy and Management, College of Public Health Science, Korea University, Seoul, Korea; 5 Department of Preventive Medicine, College of Korean Medicine, Kyung Hee University, Seoul, Korea

**Keywords:** Geriatrics, Public health, Quality of life

## Abstract

Korea is showing the fastest trend in the world in population aging; there is a high interest in the elderly population nationwide. Among the common chronic diseases, the elderly tends to have a high incidence of depression. That said, it has been vital to focus on preventing depression in the elderly in advance. Hence, this study aims to select the factors related to depression in low-income seniors identified in previous studies and to develop a prediction model. In this study, 2975 elderly people from low-income families were extracted using the 13th-year data of the Korea Welfare Panel Study (2018). Decision trees, logistic regression, neural networks, and random forest were applied to develop a predictive model among the numerous data mining techniques. In addition, the wrapper’s stepwise backward elimination, which finds the optimal model by removing the least relevant factors, was applied. The evaluation of the model was confirmed via accuracy. It was verified that the final prediction model, in the case of a decision tree, showed the highest predictive power with an accuracy of 97.3%. Second, psychological factors, leisure life satisfaction, social support, subjective health awareness, and family support ranked higher than demographic factors influencing depression. Based on the results, an approach focused on psychological support is much needed to manage depression in low-income seniors. As predicting depression in the elderly varies on numerous influencing factors, using a decision tree may be beneficial to establish a firm prediction model to identify vital factors causing depression in the elderly population.

## Introduction

The global effects of the aging population are rapidly increasing. According to the World Health Organization, the number of people aged 60 or older has quickly been increasing, and it is expected to surpass 1.4 billion by 2030 and 2.1 billion by 2050^1^. According to Statistics Korea, as of 2022, the elderly population aged 65 or older is expected to account for 17.5% of the total population; more importantly, the super-aged society is expected to reach in 2025, which accounts for 20.6% of the total population^2^. The rapidly increasing elderly population can experience various health problems due to physical and psychological changes from a life cycle point of view^3^. Therefore, Korea is showing the fastest aging population trend globally, so interest in the elderly is high nationally^4^.

Due to aging, one in four older adults experience age-related mental health issues^5^. The most common issue is known to be depression, as it may perhaps complicate an older adult’s existing health condition and trigger new concerns^6^. In the case of Korea, the number of patients with depression continues to rise, and it is expected to rank first in 2030^7^. According to the Mental Health Foundation, about 22% of the United Kingdom population aged 65 and older suffered from depression; about 28% of men and 28% of women, and about 85% of the elderly with depression had no help^8^. It has also been reported that people experiencing depression commit dangerous behaviors such as self-harm behavior to escape their negative emotions^9^. As such, depression could be related to suicide risk and is closely similar to other risk behaviors; thus, careful attention is needed in the elderly community, especially for those who have experienced depression. Based on research findings in previous studies, it was demonstrated that depression is also closely related to income level. In particular, it is reported that low-income elderly households are in worse health than other groups, and even when diagnosed with depression, the symptoms worsen because timely and appropriate treatment is not provided^10^. Therefore, it can be seen that it is more important for low-income elderly households to make efforts to detect depression early and prevent and manage it compared to other groups.

A study of income inequality, social support, and depression in older European adults found that the lower the income level corresponding to the fifth quintile was, the higher the score appeared for depression—indicating that the role of household income is essential in understanding depression^11^. Therefore, the depression of the elderly is a societal problem that cannot be overlooked as it increases the burden of caring for the person and the people around them. Since depression is a common disease in the elderly, the factors that increase depression are exceptionally diverse. Previous studies have shown that depression in the elderly is closely related to depression, including gender, age, education level, chronic disease, social support, health promotion behavior, leisure life satisfaction, and medical expenses^12,13^. Concerning elderly depression in Ethiopia, one of the low-income countries, two out of five elderly people suffer from depression. Among the elderly, those who are females, have no formal education, have chronic diseases, and have no social support, are prone to depression^14^. Notably, the lower the income level, the higher the level of depression among the elderly, which can be understood as the higher the level of depression among the elderly with a relatively high risk of exposure to economic difficulties. Other studies confirmed that income level, education level, emotional support, and subjective health awareness affected depression; moreover, it was found that the intensity of depression heightened with a low-income level, less emotional support, and low subjective health awareness^15^. In addition, the level of depression decreased when participating in satisfactory leisure activities^16^.

Based on the comprehensive literature review of the previous studies, a few studies have analyzed the differences between the factors affecting depression and the level of depression in the elderly from low-income families. Most studies were found to have a limitation in that the existing studies have only identified the factors that affect depression in an enumerated manner. Most importantly, none of the existing studies had used data mining techniques to seek models that predict depression in low-income elderly. In addition, studies using text mining methods have merely identified depressive symptoms in different groups, while only a few studies were carried out on the elderly^17–19^.

Hence, this study attempted to develop a prediction model for depression in the elderly from low-income families based on the influencing factors identified in previous studies. Data mining, a type of machine learning, was used since it is increasingly used in the healthcare field and is mainly used in developing predictive models, such as the prediction of therapeutic effects and diagnosis of hypertension and diabetes^20^. According to McKinsey and Company, data mining increases patient management efficiency, can provide correct treatment planning and diagnosis, and is of great help to complex cases^21^. The active use of data mining techniques is highly likely to reduce medical costs by up to 17%^22^. This study is expected to provide the fundamental data for developing an integrated program to prevent and manage depression in the elderly from low-income families.

## Methods

### Data resource

This study analyzed the original data of the 13th-year (2018) from the Korean Welfare Panel Survey (KOWEPS). The KOWEPS is a nationally representative longitudinal survey of 7000 households in 16 metropolitan cities, including Seoul and Jeju Island, conducted jointly by the Korea Institute for Health and Social Affairs and the Social Welfare Research Center of Seoul National University. In addition, a sample was selected by extracting a two-stage stratified cluster systematic sampling based on the income data of households in the 2006 National Welfare Survey on Living Conditions. The aforementioned households were divided into low-income and regular-income households, and 7000 households were selected using stratified cluster systematic sampling, allocating 3500 households from each group.

**Figure 1:**
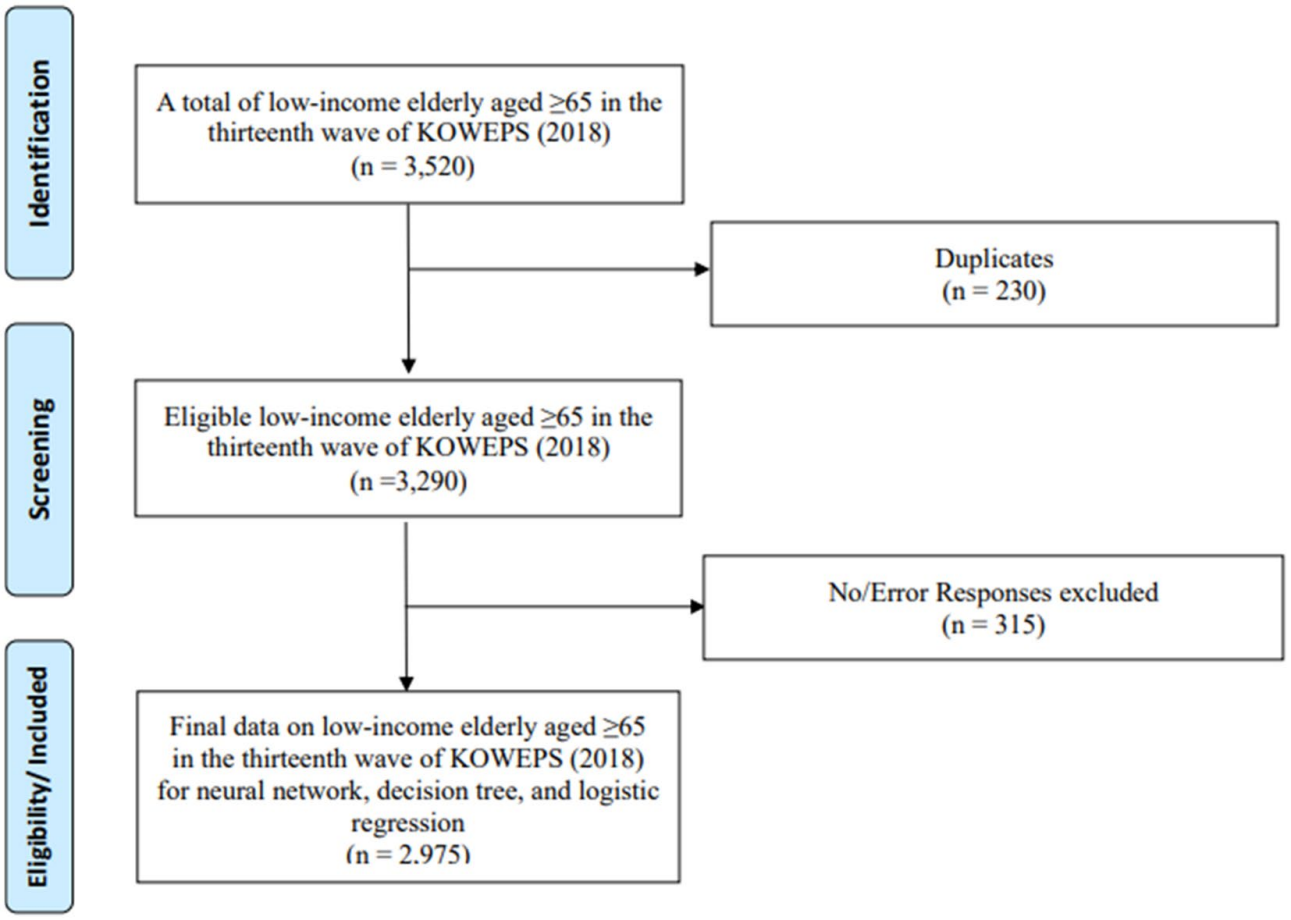
Flowchart of the study design.

The 13th-year (2018) data for household members were selected for this study since the number of elderly patients with panic disorders, non-organic sleep disorders, eating disorders, and depression increased by 81% from 290,000 in 2010 to 530,000 in 2018^23^. A total of 2975 people was analyzed, excluding missing values from the factors used in the analysis; in the final stage, data from the elderly aged 65 or older were extracted, as aged 65 or older is designed as elderly according to the welfare of senior citizens act of Korea (Fig. 1)^24^. The standard median income is the income of the person in the center of the line when all people are lined up, and the KOWEPS considers 60% or less to be low income. As aforementioned, low-income elderly are reported to have poorer health outcomes than other populations and are significantly affected by surroundings. Therefore, data from the 13th-year (2018) data was chosen, excluding any potential non-related impacts from the era of COVID-19; the first case of COVID-19 in South Korea was in January 2019^25^.

### Construction of variables

#### Target variable

The KOWEPS provides CES-D 11 (The Center for Epidemiological Studies-Depression Scale) as a measure of depression. The scale was reconstructed by reducing the 20-item instruments developed by Radloff (1977) to 11-item instruments^26^. The instruments consist of the following questions: I did not feel like eating; my appetite was poor; I felt that I was just as good as other people; I felt depressed; I felt that everything I did was an effort; My sleep was restless; I felt lonely; I enjoyed life; People were unfriendly; I felt sad; I felt that people dislike me; and I could not get “going.”

The range of responses were from 0 (rarely or none of the time) to 3 (most or all of the time). In this study, the total score of the 20-item circle scale was used for analysis by multiplying by 20/11 to determine whether or not there was depression. The higher the value, the higher the level of depression indicated. Depression can be suspected if the score is 16 point or more, and a score less than 16 can be considered normal.

#### Input variable

Based on the literature review discussed above, the input variables used in this study are as follows. Gender, age, education level, number of household members, disability, economic activity, and chronic disease were included as demographic factors. Second, social support, family support, and leisure life satisfaction are measured on a four-point Likert scale, respectively, and the higher the score, the higher the support and satisfaction. Third, health promotion behavior is a concept that encompasses various factors, such as beliefs, behaviors, and habits necessary for health promotion and maintenance. However, this study was limited to factors of health behavior and lifestyle provided by the KOWEPS. Drinking was scored as 1 point for 'the average amount of alcohol consumed per year'; if there was no drinking experience at all, 0 points for drinking experience at least once. For smoking, 'currently smoking cigarettes,' 0 points if smoking, and 1 point was given for nonsmokers. The average of the health checkup was calculated by giving 0 points if it had never been done and 1 point if it was done once; the higher the score, the more health behaviors it had. Fourth, subjective health awareness is measured on a four-point Likert scale; the higher the score, the higher the subjective health awareness, and the level of medical expenditure means the average monthly medical expenditure. The factors used in the analysis are summarized in Table 1.

**Table 1:** Variables and measurements used in the analysis.

Category	Variables	Measures
Output factor	Depression	CES-D scale 11questions (0 ~ 3 points)/Less than 16 points (0), 16 points or more (1)
13 Input factors	Sex	0 = men, 1 = women
	Age	Based on age: 0 = 65 ~ 69, 1 = 70 ~ 74, 2 = 75 ~ 79, 3 = 80 or older
	Educational level	0 = preschool, 1 = elementary school, 2 = middle school, 3 = high school, 4 = college or higher
	Number of household members	0 = 1 person, 1 = 2 people, 2 = 3 ~ 4 people, 3 = 5 or more people
	Disability	0 = no, 1 = yes
	Economic activities	0 = non-participation, 1 = participation
	Chronic disease	0 = no, 1 = yes
	Social support	0 = very dissatisfied, 1 = dissatisfied, 2 = normal, 3 = satisfied, 4 = very satisfied
	Family support	
	Leisure life satisfaction	
	Health promotion behavior (Total score)	Drinking status: 0 = yes, 1 = no Smoking status: 0 = yes, 1 = no Medical checkup: 0 = no, 1 = yes
	Subjective health awareness	0 = very poor health, 1 = not healthy, 2 = normal, 3 = good health, 4 = very good health
	Medical expenditure level	Average monthly expenditure (unit: 10,000 won)

### Statistical analysis

Frequency analysis, T-test, and one-way ANOVA analysis were performed to verify whether statistical differences occurred according to the demographic characteristics and depression level of the participants of this study. Then, data mining techniques, logistic regression analysis, decision tree analysis, artificial neural network analysis and random forest analysis were used to build a predictive model for depression in the elderly of low-income households. A sensitivity analysis was conducted to ensure that the main outcome was reliable and robust. The analysis was carried out by changing the cut-off score for suspected depression as the dependent variable.

Logistic regression analysis is the most common method used when the target factor is binary, and it has the advantage of supplementing data that only takes a value of 0–1. An artificial neural network is one of the most widely used methodologies to predict the category of target factors by combining input factors with a nonlinear model, passing them to each hidden unit, and delivering the combination of hidden units to the output node. A decision tree analysis is a technique that classifies the categories of target factors by tabulating decision-making rules in the form of a tree structure. Since it is expressed in a tree structure, it is easy to interpret the classification results and has the advantage of obtaining information on major predictive factors. In this study, C5.0, one of the types of decision trees, was used. Random forest is a model that improves the shortcomings of decision tree and is reported to have excellent performance because it can prevent overfitting by applying bagging technique to generate multiple decision trees^27^. Finally, logistic regression analysis was conducted to identify the predictors of high risk of depression. For the development and evaluation of the predictive model, a tenfold cross-validation method was used in which the entire data was divided into ten categories for generalization and used as model creation (9) and validation (1) data^28^. After examining the relative importance of predictive factors via Shapley additive explanation analysis that contributed to predicting the depression level of the elderly in low-income households, wrapper's stepwise backward elimination was applied to find the optimal model by removing the least relevant factors. The models created via the process mentioned above were evaluated based on accuracy, and then the optimal model for this topic was selected.

The performance index of the developed prediction model means that the larger the size, the stronger the predictive power of the depression level. The model's final evaluation was based on accuracy, and sensitivity and specificity values were also presented. The analysis packages, IBM SPSS Modeler 18.0 (SPSS Inc., Chicago, Illinois, USA) and SAS 9.4 (SAS Institute Inc., Cary, NC), were used.

### Ethical approval

This study was approved by the Korea University Institutional Review Board (IRB No. IRB-2022-0385). The IRB of Korea University waived informed consent since this study was retrospective and blinding of the personal information in the data was performed.

## Results

The results of the demographic characteristics of the study and the average difference in depression levels are shown in Table 2. Females accounted for a higher number than males; females (n = 2008, 67.5%) and males (n = 967, 32.5%). For the age distribution, ‘ages of 80 or older’ was the largest with 1475 people (49.6%), followed by ‘ages of 75–79’ with 755 people (25.4%) and ‘ages of 70–74’ with 459 people (15.4%). Regarding the level of education, 2107 people (70.8%) had ‘elementary school graduation’, and 471 people (15.8%) had ‘middle school graduation’. indicating that the majority had a low level of education. When asked about the number of household members, ‘two people’ accounted for the largest portion with 1459 people (49.0%), showing that the majority lived with one more person. As for having disabilities, 2425 people (81.5%) mentioned ‘no’, and 550 people (18.5%) stated ‘yes’. In terms of participation in economic activities, ‘not participating’ accounted for more than half of the participants (n = 2042, 68.6%).

**Table 2:** General characteristics of the participants and differences in depression level.

Variables		N	%	*t or F*
Sex	Men	967	32.5	− 3.547***
	Women	2008	67.5	
Age	65–69	286	9.6	− 2.997
	70–74	459	15.4	
	75–79	755	25.4	
	80 or older	1475	49.6	
Educational level	Elementary school	2107	70.8	1.969
	Middle school	471	15.8	
	High school	310	10.4	
	College or higher	87	2.9	
Number of Household members	1	1292	43.4	11.997
	2	1459	49.0	
	3 or more people	224	7.5	
Disability	Yes	550	18.5	− 1.52
	No	2425	81.5	
Economic activities	Yes	933	31.4	7.326***
	No	2042	68.6	
Depression	Yes	422	14.2	–
	No	2553	85.8	

p* < .05, p** < .01, p*** < .001.

In terms of depression, 2553 people (85.8%) reported ‘no’ and 422 people (14.2%) reported ‘yes.’ Lastly, the average difference between the sociodemographic characteristics and the depression level of the participants was evaluated. As a result, there were significant gender differences (t = − 3.547, *p* < 0.001) and participation in economic activities (F = 7.326, *p* < 0.001), but no differences were found in other factors.

The descriptive statistical results of the main factors are shown in Table 3. Considering that the range of scores for health promoting behavior is from a minimum of 0 to a maximum of 3, an average of 2.2 points can be regarded as a high value. On the other hand, having a standard deviation of 0.72, it can be understood that there was no significant difference in health promotion behavior by the elderly in low-income households. With reference to subjective health awareness, it was found that numerous elderly people had a higher awareness than the average, with an average of 2.8 points. Regarding the level of medical expenses, it was found that the average monthly expenditure was 158,000 won, and the standard deviation was 20.17, indicating a high difference in expenditure among the elderly in low-income households. Family support, social support, and leisure life satisfaction showed average scores of 2.7, 2.6, and 2.3, respectively, which were verified to be in good standing, considering that the range of scores was at least 0 to up to 4.

**Table 3:** Descriptive analysis of major variables.

Variable	Mean	Standard Deviation	Range
Health promotion behavior	2.2	.72	0–3
Subjective health awareness	2.8	.85	1–5
Medical Expenditure Level (Month)	15.8	20.17	0–196
Family support	2.7	.67	0–4
Social support	2.6	.64	0–4
Leisure life satisfaction	2.3	.73	0–4

**Figure 2:**
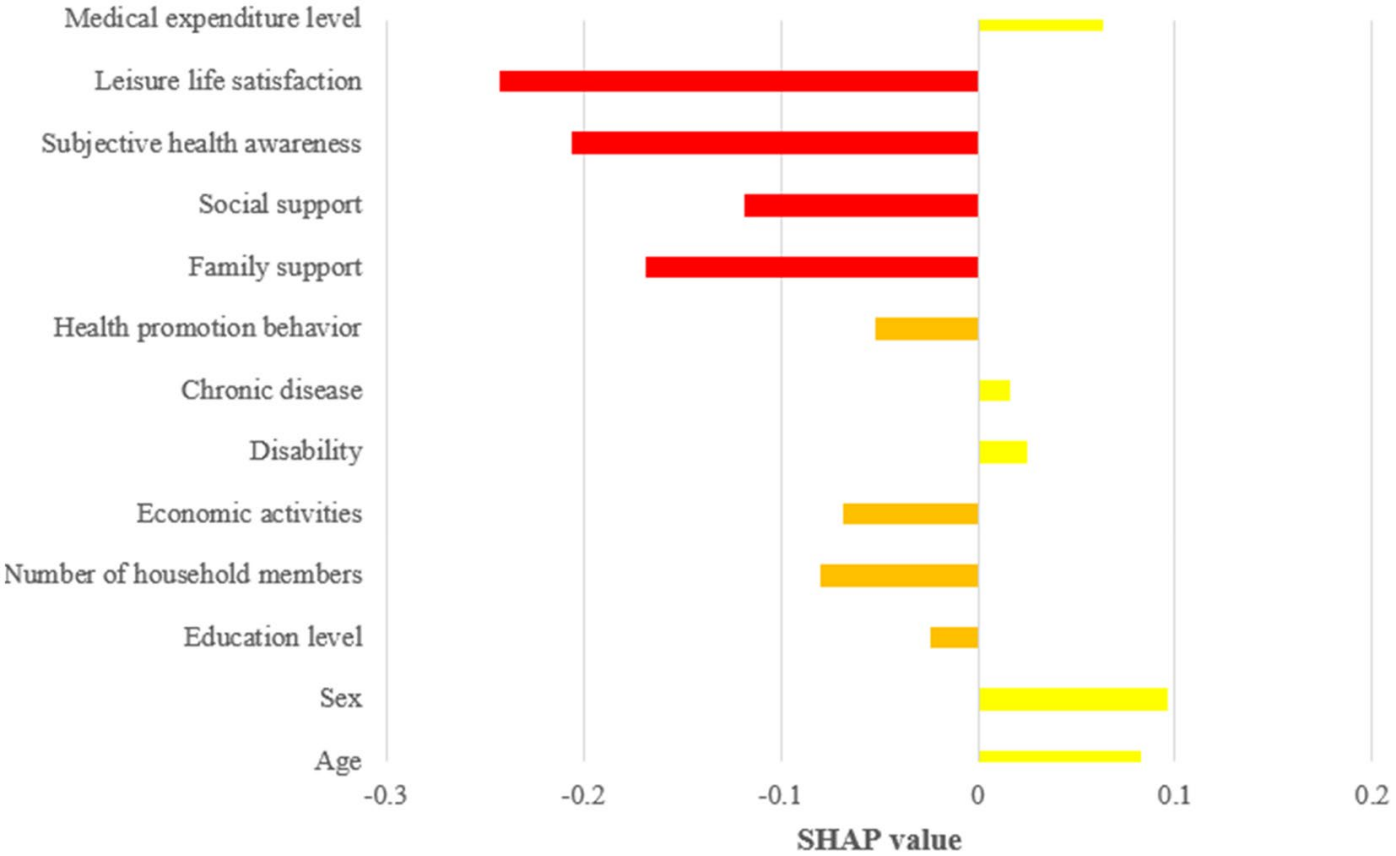
The summary plot of the SHAP values.

The relative importance of the predictive factors that contributed to predicting depression in low-income seniors utilizing the feature selection, is shown in Table 4. The higher the order of importance of a predictor, the greater the influence of that factor in predicting the level of depression; the highest ranking was identified as 'leisure life satisfaction.' This result can be interpreted as having the greatest effect on satisfaction in leisure life than other factors when predicting the level of depression of the elderly in low-income households. Furthermore, the factors of subjective health awareness, family support, and social support were found to be in the upper ranks. However, it was noted that the factors of presence or absence of chronic diseases, educational level, disability, and health behavior were distributed in the low ranking. A SHAP summary plot was created (Fig. 2), a visualization of how much each explanatory variable affects the prediction of depression. A yellow bar indicates a positive influence on the occurrence of depression. The red and orange bars indicate a negative impact on the occurrence of depression. The red bars were found to be the most influential variables. Regarding leisure life satisfaction, it can be used as an explanatory or a dependent variable. This study used it as an explanatory variable because the subjects were low-income elderly. The relationship between leisure life satisfaction and depression in low-income elderly is often reported as causal, with leisure life satisfaction affecting depression^29^.

**Table 4:** The importance of variables that affect the level of depression.

Variables	Ranking	Variables	Ranking
Leisure life satisfaction	1	Economic activities	8
Subjective health awareness	2	Medical Expenditure Level	9
Family support	3	Health behavior	10
Social support	4	Disability	11
Sex	5	Educational level	12
Age	6	Chronic disease	13
Household members	7

In this study, the classification techniques used to develop the most accurate predictive model, predicting the level of depression of the elderly in low-income households, were artificial neural networks, decision trees, logistic regression and random forest analysis. Table 5 is the result of the classification analysis by sequentially applying the wrapper's stepwise method to the relative importance of the factors identified in Table 4. Based on the analysis, it was identified that the decision tree algorithm showed higher predictive power than the other three algorithms. In the case of logistic regression analysis, the prediction accuracy was 73.2%, and the artificial neural network showed 81.8%. On the other hand, the decision tree shows a tendency to increase predictive accuracy as the number of factors increases, except when there is only one input factor. When all 13 factors were input, an accuracy of 97.3%, a sensitivity of 100%, and a specificity of 94.6% were presented. Finally, when forming the decision-making tree, the factor that had the greatest impact was the subjective health awareness factor, followed by leisure life satisfaction, family support, and social support. To ensure that the main outcome was reliable and robust, a sensitivity analysis was conducted by dividing the dependent variable, depression incidence, into two thresholds (15 points or less, 16 points or more); the analysis revealed that the main outcome did not change in Tables 6, 7.

**Table 5:** Distribution of accuracy, sensitivity, and specificity by models.

No.	NN			DT			LR			RF		
	Accuracy	Sensitivity	Specificity	Accuracy	Sensitivity	Specificity	Accuracy	Sensitivity	Specificity	Accuracy	Sensitivity	Specificity
1	68.9	51.9	86.2	67.6	46.5	86.8	67.3	48.2	86.4	66.4	68.2	75.9
2	70.0	77.8	61.5	71.5	80.7	62.2	70.1	77.5	63.2	71.1	78.5	74.1
3	74.1	75.7	72.5	76.0	73.7	78.3	75.1	79.5	71.0	73.8	76.4	80.3
4	77.1	77.7	76.6	81.5	85.2	77.6	75.6	75.5	75.7	75.4	80.3	81.9
5	76.8	75.0	78.5	82.7	87.4	78.0	69.7	71.4	68.0	77.5	82.9	82.1
6	76.4	76.5	75.2	83.0	87.5	78.7	73.8	75.0	71.7	77.2	85.1	79.3
7	78.6	71.9	78.0	87.3	90.7	83.5	75.1	76.5	73.8	75.2	79.5	72.3
8	79.5	76.5	75.2	83.0	87.5	78.7	73.3	75.0	72.7	79.2	77.2	81.9
9	79.0	76.9	81.4	92.3	97.8	85.9	75.9	76.5	75.2	85.8	78.2	82.1
10	80.2	77.8	83.8	92.8	98.7	87.1	76.3	75.3	77.3	86.2	82.6	80.0
11	80.7	83.0	78.7	94.5	100	90.1	75.7	76.8	74.8	89.2	82.5	79.8
12	81.2	81.4	81.1	97.3	100	92.7	74.3	71.2	75.3	87.3	84.2	80.0
13	81.8	85.5	77.8	97.3	100	94.6	73.2	71.8	76.5	88.5	86.2	84.3

NN: neural network, DT: decision tree, LR: logistic regression, RF: random forest.

**Table 6:** Sensitivity analysis results (depression level: 15 point or less).

No.	NN			DT			LR			RF		
	Accuracy	Sensitivity	Specificity	Accuracy	Sensitivity	Specificity	Accuracy	Sensitivity	Specificity	Accuracy	Sensitivity	Specificity
1	70.2	65.2	70.2	74.1	72.0	80.6	64.6	65.9	70.6	74.6	77.9	76.2
2	72.1	73.2	65.2	77.9	80.5	79.5	65.8	70.2	69.2	77.2	80.2	76.8
3	76.2	74.2	72.9	76.2	79.2	81.5	71.9	75.5	74.5	78.8	81.2	83.2
4	74.2	76.5	74.2	80.2	82.1	84.5	74.3	76.5	79.5	81.0	85.4	80.4
5	78.6	79.5	86.5	84.5	86.5	86.5	76.4	75.6	80.6	84.3	87.2	81.9
6	77.5	77.9	80.2	88.5	89.6	89.5	78.6	79.9	76.5	86.2	88.6	85.6
7	79.2	80.2	84.5	87.2	85.5	87.2	78.8	75.9	79.5	87.2	89.5	92.5
8	80.9	85.9	88.5	89.2	92.5	91.9	79.2	74.9	81.5	88.9	90.6	84.2
9	82.4	84.2	89.2	90.2	94.2	90.2	82.1	80.9	84.5	91.5	91.5	89.2
10	85.1	86.2	91.0	92.5	96.5	91.2	83.4	74.2	85.4	90.2	92.4	87.0
11	88.9	90.8	87.2	96.2	100	95.2	85.3	78.3	91.8	93.1	87.2	89.2
12	87.9	86.6	89.0	94.2	100	96.6	87.0	80.8	92.7	90.9	89.2	93.5
13	88.8	85.5	91.2	95.3	100	96.5	84.0	78.0	90.1	92.4	95.1	90.2

NN: neural network, DT: decision tree, LR: logistic regression, RF: random forest.

**Table 7:** Sensitivity analysis results (depression level: 16 point or more).

No.	NN			DT			LR			RF		
	Accuracy	Sensitivity	Specificity	Accuracy	Sensitivity	Specificity	Accuracy	Sensitivity	Specificity	Accuracy	Sensitivity	Specificity
1	66.4	62.1	69.9	75.9	82.1	84.0	62.1	68.9	68.1	74.2	78.2	79.5
2	68.5	72.1	72.1	80.2	86.4	86.4	65.4	69.2	69.8	76.5	72.1	82.6
3	70.4	69.4	74.2	85.6	89.4	89.5	67.8	71.5	72.9	78.9	77.5	86.8
4	73.9	74.5	79.2	87.6	88.9	88.6	69.8	70.2	74.1	80.5	76.5	84.5
5	72.5	76.5	75.5	89.4	90.5	90.5	70.5	72.1	75.1	81.9	89.9	88.4
6	75.4	79.5	79.2	88.4	91.5	91.5	75.1	76.5	70.5	83.9	86.4	89.8
7	76.4	76.8	75.4	90.2	94.2	92.4	72.1	75.4	76.0	84.4	90.1	90.5
8	79.2	77.6	77.6	93.5	97.5	96.5	74.5	79.5	77.9	87.1	93.1	93.5
9	78.2	80.5	81.2	94.5	98.4	95.4	76.5	76.8	78.5	89.4	95.4	94.2
10	80.2	79.8	80.7	97.1	100	96.4	77.2	77.5	77.2	87.2	92.1	93.1
11	77.6	78.0	77.1	96.1	100	95.2	77.1	80.0	74.1	84.6	89.1	90.2
12	82.8	88.5	77.0	96.5	100	96.7	78.2	78.1	78.4	85.4	90.2	89.8
13	83.2	85.3	81.0	95.4	100	94.2	74.1	74.7	73.5	85.6	88.5	91.2

NN: neural network, DT: decision tree, LR: logistic regression, RF: random forest.

Logistic regression analysis was performed to seek the influence of the predictors of high risk of depression in the elderly from low-income households, and the results are shown in Table 8. The factors that affected the level of depression were gender, number of household members, subjective health awareness, family support, social support, and satisfaction with leisure life. In the case of gender, the probability of developing depression in women was confirmed to be 1.86 times (OR = 1.861, 95% CI = 1.173–2.954) higher than in men. As the number of household members increased by each level, the probability of depression decreased by 0.69 times (OR = 0.692, 95% CI = 0.513–0.933). In subjective health awareness, an increase of each level was associated with a 0.40-fold (OR = 0.403, 95% CI = 0.312–0.522) lower probability of depression. Further, family support (OR = 0.613, 95% CI = 0.494–0.759), social support (OR = 0.711, 95% CI = 0.552–0.916), and leisure life satisfaction (OR = 0.425, 95% CI = 0.328–0.425) showed that the probability of depression decreased by 0.61 times, 0.71 times, and 0.42 times, respectively, as the level increased by each level.

**Table 8:** The results of logistic analysis according to the level of depression of the elderly in low-income households.

Variables	OR	95% CI		*p*-value
		Lower	Upper	
Age	1.005	.979	1.033	.688
Sex (Ref: men)	1.861	1.173	2.954	.008**
Educational level	.954	.785	1.160	.638
Household members	.692	.513	.933	.016*
Economic activities (Ref: no)	.598	.357	1.002	.051
Chronic disease (Ref: yes)	.858	.563	2.653	.612
Health promotion behavior	.801	.628	1.022	.074
Subjective health awareness	.403	.312	.522	.000***
Medical expenditure level	1.004	.996	1.011	.337
Family support	.613	.494	.759	.000***
Social support	.711	.552	.916	.008**
Leisure life satisfaction	.425	.328	.425	.000***

p* < .05, p** < .001, p*** < .001, OR: odds ratio, CI: confidence interval.

## Discussion

This study analyzed the factors affecting the depression of the elderly from low-income families, using the KOWEPS data based on the literature review mentioned above. The study initially determined whether the factors are related to depression in the elderly of low-income families and then developed a prediction model to predict depression. As a result of the analysis, the decision tree had the highest accuracy as a model for predicting depression among the elderly from low-income families, and the factors that greatly influenced the formation of the model were mainly psychological.

The main findings are as follows. First of all, as a result of sequentially applying wrapper's step-by-step removal method to the relative importance of factors that affect predicting depression in the elderly from low-income families, it was confirmed that the decision tree analysis showed the highest predictive power (97.3%). This result is consistent with previous studies that decision trees show excellent results in developing predictive models. As Lee et al., stated, when developing a model that predicts patient satisfaction and revisits intention according to hospital visits, artificial neural networks, logistic regression analysis, and decision trees (C5.0, CART, QUEST) were used, and the decision trees showed the highest predictive power, and C5.0 showed excellent results^30^. Moreover, decision trees (C5.0, CHAID, and QUEST) were used in a model development study that predicts whether patients with severe work histories are admitted to the intensive care unit. As a result, it was found that C5.0 showed the best predictive power^31^. With all that said, the decision tree (C5.0) has the advantage of having an algorithm that can more effectively handle complex relationships between predictors, which is widely used in the healthcare field. More importantly, it is known as one of the classification techniques of data mining with proven effectiveness^32^. It is expected that effective depression management services can be provided by detecting groups with a high risk of depression at an early stage. Further refinement of the model to include additional community infrastructure and geographic factors related to depression may lead to more diverse measures to prevent depressive problems among low-income elderly.

Second, when the decision tree (C5.0) was formed, subjective health awareness, leisure life satisfaction, family support, and social support were the factors that had a relatively significant influence. This outcome is supported by a study that depressive disorder in the elderly is on the rise worldwide and that psychological factors such as social support and subjective health awareness are key contributing factors^33^. Another study reported that life satisfaction and subjective health awareness have the most significant influence^34^. Depression in the elderly has been shown to have a significant psychological impact, and decision trees are reported to be a highly effective method^35,36^. In order to prevent and manage depression in the elderly, it is necessary to recognize the need for policy support considering psychological factors (subjective health awareness, leisure life satisfaction, family support, and social support). For example, adequate mental health management can be provided by conducting free quarterly psychological examinations on low-income elderly at public health centers and local clinics in each region to detect risk groups for depression while developing and operating programs to increase psychological support in the community service centers.

Third, a logistic regression analysis was conducted to confirm the predictors of depression in the low-income elderly. As a result, gender, the number of household members, subjective health awareness, leisure life satisfaction, family support, and social support were identified as influencing factors. It was found that the higher the risk of depression, especially for women, the smaller the number of household members, the lower the satisfaction level of leisure life, the lower the family support and social support, and the lower the level of subjective health awareness. These results were aligned with the same context as previous studies^18,35–37^. The level of depression according to income level can also be examined. Muhammad et al. reported that the elderly population in the poorest fifth quintile was 39% more likely to develop depression than the elderly in the first quintile^38^. Thus, it can be presumed that depression in the elderly is not caused by a single factor but by a combination of various factors. With that mentioned, forming activities in the local community that senior citizens can participate in, such as senior universities and clubs, while encouraging active promotion and participation are considered to prevent depression in the long run. All activities could be provided free of charge considering the characteristics of the low-income elderly, and if necessary, it may be an idea to encourage participation by offering a subsidy. In South Korea, various psychological support programs for the elderly exist in different regions so that it would be more effective to form a network to establish and manage roles and functions across regions. For example, the community service centers in each region act as gatekeepers to identify groups of people who are likely to be depressed and encourage to participate in the community-based psychological support program.

Finally, the limitations of this study are as follows. First, various factors affecting depression in the elderly were not examined. Previous studies have shown that various factors, such as biological factors, cultural factors, and environmental factors, act in combination to affect depression; however, the current study did not include all factors due to data limitations. Second, this study was conducted as a cross-sectional study, and there are some difficulties in identifying the causal relationship over time. Thirdly, in terms of the influence of depression, the characteristics of the age of the elderly were not considered. Since recent old age has various characteristics by period, which are classified into the first, middle, and late stages, it is highly likely that different patterns will appear regarding the factors influencing depression and the size of its impact.

## Conclusion

This study selected factors related to depression in the elderly from low-income families identified in previous studies to develop a prediction model considering depression in the elderly from low-income families. As a result of the study, psychological factors (leisure life satisfaction, subjective health awareness, family support and social support) were higher than demographic factors, and the most suitable predictive model was identified as a decision tree. The aforementioned results suggest that an approach focused on psychological support is needed to manage the level of depression in low-income seniors. More importantly, as several influencing factors of depression vary in the elderly population, utilizing a decision tree will be beneficial to establish a more concrete prediction model.

## Data Availability

The data can be available for a special purpose in request to the first author of the study.

## Abbreviations

CES-D: The Center for Epidemiological Studies-Depression Scale
CI: Confidence Interval
COVID-19: Corona Virus Disease 19
DT: Decision Tree
KHPS: Korea Health Panel Study
LR: Logistic Regression
NN: Neural Network
OR: Odds Ratio
RF: Random Forest
SHAP: Shapley Additive Explanation
